# The cholesterol synthesis enzyme lanosterol 14α-demethylase is post-translationally regulated by the E3 ubiquitin ligase MARCH6

**DOI:** 10.1042/BCJ20190647

**Published:** 2020-01-31

**Authors:** Nicola A. Scott, Laura J. Sharpe, Isabelle M. Capell-Hattam, Samuel J. Gullo, Winnie Luu, Andrew J. Brown

**Affiliations:** School of Biotechnology and Biomolecular Sciences, UNSW Sydney, Sydney, New South Wales 2052, Australia

**Keywords:** cholesterol, cytochrome P450, DHCR24, lanosterol 14α-demethylase, MARCH6, protein turnover

## Abstract

Cholesterol synthesis is a tightly controlled pathway, with over 20 enzymes involved. Each of these enzymes can be distinctly regulated, helping to fine-tune the production of cholesterol and its functional intermediates. Several enzymes are degraded in response to increased sterol levels, whilst others remain stable. We hypothesised that an enzyme at a key branch point in the pathway, lanosterol 14α-demethylase (LDM) may be post-translationally regulated. Here, we show that the preceding enzyme, lanosterol synthase is stable, whilst LDM is rapidly degraded. Surprisingly, this degradation is not triggered by sterols. However, the E3 ubiquitin ligase membrane-associated ring-CH-type finger 6 (MARCH6), known to control earlier rate-limiting steps in cholesterol synthesis, also control levels of LDM and the terminal cholesterol synthesis enzyme, 24-dehydrocholesterol reductase. Our work highlights MARCH6 as the first example of an E3 ubiquitin ligase that targets multiple steps in a biochemical pathway and indicates new facets in the control of cholesterol synthesis.

## Introduction

Dysregulation of cholesterol homeostasis leads to numerous diseases including cardiovascular disease [1], cancers [2], neurological disorders [3] and inborn developmental disorders [4]. Cholesterol synthesis is, therefore, a tightly regulated process. Cholesterol synthesis occurs via the mevalonate pathway which involves over 20 enzymes and two parallel branches: the Bloch and Kandutsch–Russell pathways. There are two established rate-limiting enzymes in cholesterol synthesis: 3-hydroxy-3-methylglutaryl-CoA reductase (HMGCR) [5,6] and squalene monooxygenase (SM) [7], which are rapidly degraded in response to sterols. The terminal enzymes in the pathway have been investigated, with 7-dehydrocholesterol reductase (DHCR7) being regulated in a similarly sterol-responsive manner [8], whilst 24-dehydrocholesterol reductase (DHCR24) remains stable [9]. However, little work has been conducted on the intermediate enzymes in the pathway, including lanosterol synthase (LSS) and lanosterol 14α-demethylase (LDM, also known by its gene name CYP51A1) which immediately follow SM, and the sterol isomerase emopamil-binding protein (EBP), which acts at the branch point to the modified Kandutsch–Russell pathway [10]. Here, we focus particularly on LDM.

LDM is found in all biological kingdoms and is considered the most ancient cytochrome P450 [11]. Despite low sequence homology, the structure and mode of action of LDM is evolutionarily conserved. LDM is responsible for three sequential monooxygenation events to catalyse the 14α-demethylation of lanosterol to form follicular fluid meiosis-activating sterol (FF-MAS) in the Bloch pathway, or 24,25-dihydrolanosterol to form dihydro-FF-MAS in the Kandutsch–Russell pathway [12]. Dysfunction of LDM has been implicated in the developmental disorder Antley–Bixler syndrome [13] and may lead to liver cirrhosis and the formation of cataracts [14–16]. In addition, LDM is responsible for the production of sterols which may activate meiosis and gamete formation [17,18], although this remains disputed [19]. Furthermore, LDM (or its yeast equivalent, Erg11p) is a drug target in infectious diseases such as fungal infections [20], Chagas disease [21], leishmaniasis [22] and tuberculosis [23]. LDM, therefore, has important biological consequences within the sterol synthesis pathway [24].

The transcription of *CYP51A1* is regulated by multiple factors including sterol-regulatory element-binding protein-2 (SREBP-2), cyclic-AMP [25,26] and hypoxia [27]. However, little work has been conducted on the post-translational regulation of LDM. A recent paper reported that LDM undergoes proteasomal degradation, triggered by nitric oxide [28]; however, the proteins involved in this degradation process have yet to be uncovered. The key signal for proteasomal degradation of a substrate is the attachment of ubiquitin via an E3 ubiquitin ligase [29]. Erg11p (yeast LDM) is degraded by the Asi E3 ubiquitin ligases [30], which do not have equivalent human homologues. The E3 ubiquitin ligase for LDM has yet to be identified. Possible human candidates for LDM include the E3 ubiquitin ligases Hrd1 and membrane-associated ring-CH-type finger 6 (MARCH6) as they target cholesterol synthesis enzymes [31,32], and gp78 and CHIP as they target other cytochrome P450s for degradation [33]. MARCH6 is involved in the degradation of SM and HMGCR [31], other sterol and lipid metabolism substrates [34–36], and is itself stabilised by cholesterol [37], indicating an important role in cholesterol homeostasis.

We primarily aimed to investigate the post-translational regulation and degradation of LDM. We identified similar transcriptional regulation of *LSS* and *CYP51A1* by sterol status, but distinct differences in their post-translational regulation. LSS remained stable, whilst LDM underwent relatively rapid degradation. Furthermore, this degradation is not triggered by sterols but does occur through MARCH6. We have also implicated MARCH6 in the degradation of the terminal enzyme DHCR24, cementing the importance of MARCH6 in the regulation of sterol synthesis.

## Materials and methods

### Plasmids

The protein-coding sequences of *CYP51A1* and *EBP* were amplified from HeLaT cDNA and cloned into our in-house pcDNA5/FRT construct [38] with a C-terminal V5 tag, with either a CMV or TK promoter. The pcDNA5-DHCR24-V5/FRT [39], pcDNA5-MARCH6-myc/FRT, pcDNA5-MARCH6-C9A-myc/FRT [31] were previously generated. The pEF1a-HA-Ubiquitin was a kind gift from Dr. Bao-Liang Song (Wuhan University, China) [40]. The empty vector plasmid, pcDNA5-EV, was used to equalise transfections.

### Cell culture

Chinese hamster ovary-7 (CHO-7) cells (kind gifts of Drs. Brown and Goldstein, University of Texas Southwestern) stably expressing LSS with a myc epitope tag (CHO-LSS-myc) [41] or DHCR24 with a V5 epitope tag (CHO-DHCR24-V5) were previously generated [39]. CHO-7 cells stably expressing LDM with a V5 epitope tag (CHO-LDM-V5) or EBP with a V5 epitope tag (CHO-EBP-V5) were generated using CHO-7 FRT cells as described previously [39]. CHO cells were maintained in DMEM/F12 medium supplemented with 5% (v/v) lipoprotein-deficient newborn calf serum (LPDS). HeLaT cells were maintained in RPMI medium supplemented with 10% (v/v) foetal calf serum (FCS). Be(2)C, HepG2 and HuH7 cells were maintained in DMEM with high glucose supplemented with 10% (v/v) FCS. All maintenance media were supplemented with penicillin (100 units/ml) and streptomycin (100 μg/ml).

Cells were seeded in 6-well or 12-well plates, transfected the following day if required and/or treated as described in the figure legends. Treatments were carried out in sterol-depleted media (LPDS for CHO-7 cell lines or foetal calf LPDS (FCLPDS) for HeLaT, Be(2)C and HuH7 cell lines) unless otherwise indicated.

### Plasmid and siRNA transfections

Prior to transfection, various CHO-7 cell lines were refreshed with DMEM/F12 medium supplemented with 5% (v/v) LPDS without antibiotics.

For plasmid transfections, CHO-LSS-myc cells were seeded into a 6-well plate and transfected the next day with 1 μg DNA and 3 μl Lipofectamine LTX transfection reagent (Invitrogen) for 24 h, then treated as indicated in the figure legends.

For immunoprecipitation experiments, CHO-7 cells were seeded into 10 cm dishes and transfected the next day with 5.8 μg DNA (2.3 μg pcDNA5-DHCR24-V5/FRT or pcDNA5-LDM-V5/FRT, 2.3 μg pcDNA5-MARCH6-myc/FRT or pcDNA5-MARCH6-C9A-myc/FRT, 1.2 μg pEF1a-HA-Ubiquitin, and pcDNA5-EV to make up to total amount when necessary), 11.6 μl P3000 supplemental reagent and 8.7 μl Lipofectamine 3000 transfection reagent (Invitrogen) for 24 h.

For siRNA transfections, CHO-LSS-myc, CHO-LDM-V5, CHO-EBP-V5 or CHO-DHCR24-V5 cells were seeded into a 6-well plate and transfected with 25 nM siRNA (MARCH6: [31], Hrd1: CTGTACATGGCCTTCATGA, gp78: GTCCTGTCTCTGTTGATTG and CHIP: GAGATATCCCTGACTACTT) using 5 μl Lipofectamine RNAiMAX transfection reagent (Life Technologies) for 24 h. Cells were harvested or treated as indicated in the figure legends for Western blotting or quantitative real-time PCR (qRT-PCR).

### Quantitative real-time PCR (qRT-PCR)

Total RNA was harvested in Tri-Reagent and reverse transcribed to cDNA using Superscript III reverse transcriptase (Invitrogen). qRT-PCR was carried out using the SensiMix SYBR Green PCR master mix (Bioline) and gene expression levels of *SQLE* (F: CAGCTCGAGGCCAGGAGG, R: CATGATAACCACCCGGCTGCAGG), *LSS* (F: AGCTGGCTGCTCCGCGTGG, R: GCTCCTGGAAGGCAGTGGAGGC), human *CYP51A1* (F: GGCTCTTACCAGGTTGGCTGCC, R: GTTGAGGATGTATGCTGCCCTGCC), total human and hamster *CYP51A1* (F: CCTGAYCGMTACTTACAGGATAACCCAGC, R: RTATCCATYRATGAGRTCAAATTCATATAAACGAAGC), *Hrd1* (F: ACATGCTCTACACGGAGCTG, R: CAGGGCAGTCTCTTAGCACC), *MARCH6* ([31]), *Gp78* (F: TACACAGGCAAGCAACTCCC, R: CACGCTCCGTCTGAAGAGAA) and *CHIP* (F: TGTGTGGCAAGATCAGCTTTGAGC, R: CCCAGCCATTCTCAGAGATGAATGC) were determined and normalised to the housekeeping gene, *porphobilinogen deaminase*(*PBGD*) [42], using the ΔΔ*C_T_* method.

### Immunoprecipitation

Following transfection, CHO-7 cells were treated with a proteasomal inhibitor (50 μM MG132) in DMEM/F12 medium supplemented with 5% (v/v) LPDS, penicillin (100 units/ml) and streptomycin (100 μg/ml) for 6 h. Cells were washed twice, then scraped in cold PBS and pelleted via centrifugation at 2500 ***g*** for 5 min at 4°C. Pellets were lysed in RIPA buffer (20 mM Tris–HCl (pH 7.4), 0.1% (w/v) SDS, 1% (v/v) Nonidet P-40, 0.5% sodium deoxycholate, 150 mM NaCl, 5 mM EDTA and 1 mM sodium orthovanadate) containing 10 mM *N*-ethylmaleimide, 50 μM MG132 and cOmplete^TM^ ULTRA Protease Inhibitor Mixture Tablets (one tablet per 10 ml of RIPA buffer). Lysates were passed through a 23-gauge needle 20 times, before centrifugation at 17 000 ***g*** for 15 min at 4°C. The protein concentration of the supernatant was determined using a Pierce Bicinchoninic Acid Protein Kit (Thermo Fischer Scientific), normalised across conditions, and then precleared with normal mouse IgG (Santa-Cruz Biotechnology) conjugated to magnetic Protein G Dynabeads. The supernatant was then immunoprecipitated overnight at 4°C with anti-V5 antibody (5 μg) conjugated to magnetic Protein G Dynabeads. Beads were then washed three times in RIPA buffer by rotating at 4°C. The bound proteins were then eluted by heating at 65°C for 15 min in elution buffer (1× Laemmli sample buffer, 0.4× RIPA buffer, 4% (w/v) SDS), before being subjected to Western blotting.

### Western blotting

Protein lysate was harvested in 3% SDS lysis buffer (3% (w/v) SDS, 10 mM Tris–HCl, pH 7.6, 100 mM NaCl) with 2% (v/v) protease inhibitor cocktail (Sigma–Aldrich), passed through a 23-gauge needle 20 times and vortexed for 20 min. Protein concentration was determined using a Pierce Bicinchoninic Acid Protein Kit (Thermo Fischer Scientific) and normalised in lysis buffer with 1× Laemmli buffer. Normoxic and hypoxic lysates from HeLaT and HepG2 cells were obtained from Novus Biologicals. Equal amounts of protein were subjected to 10% (w/v) SDS–PAGE and transferred to nitrocellulose membranes (Bio-Rad). Membranes were blocked in 5% (w/v) skim milk in PBST and probed with the following primary antibodies: mouse anti-V5 tag (1 : 5000; Life Technologies), mouse anti-myc tag (1 : 1000; Santa Cruz Biotechnology), rabbit anti-myc tag (1 : 2000; Abcam), rabbit anti-HA tag (1 : 2000; Cell Signalling Technology), Penta-His HRP (1 : 10 000; Qiagen), rabbit anti-LDM (1 : 5000; ProteinTech), rabbit anti-SM (1 : 5000; ProteinTech), mouse anti-α-tubulin (1 : 10 000; Sigma–Aldrich) or rabbit anti-vinculin (1 : 5000; Abcam). After incubation with primary antibodies, blots were incubated with either IRDye® 680RD donkey anti-rabbit (1 : 10 000; LI-COR Biosciences) and IRDye® 800CW donkey anti-mouse (1 : 10 000; LI-COR Biosciences) or HRP donkey anti-rabbit or donkey anti-mouse secondary antibodies (Jackson ImmunoResearch Laboratories) for 1 h, and then imaged accordingly: LI-COR secondary antibody blots were imaged using the Odyssey CLx system (LI-COR Biosciences) and HRP secondary antibody blots were imaged using the ImageQuant LAS 500 (GE Healthcare). Protein band intensities were quantified by densitometry using Image Studio Lite (version 5.2).

### Data presentation and statistical analysis

qRT-PCR data are representative of at least three independent experiments. Data are presented in the form of bar graphs as mean + SEM. Statistical testing was performed using Student's paired *t*-test (two-tailed), where *P-*values of < 0.05 (*) and < 0.01 (**) were considered statistically significant.

To determine relative protein levels, treated samples were normalised to control conditions, which were set to 1. For data presented as percentage protein remaining, the ratio between the two conditions at each time point was calculated. All Western blots are representative of at least three independent experiments, except for the commercial normoxic/hypoxic lysates. Densitometry data are presented as bar graphs or line graphs with mean ± SEM. Statistical testing was performed using Student's paired *t*-test (two-tailed), where *P-*values of < 0.05 (*) and < 0.01 (**) were considered statistically significant.

## Results

### *LSS* and *CYP51A1* are transcriptionally responsive to sterols

**Figure 1.**
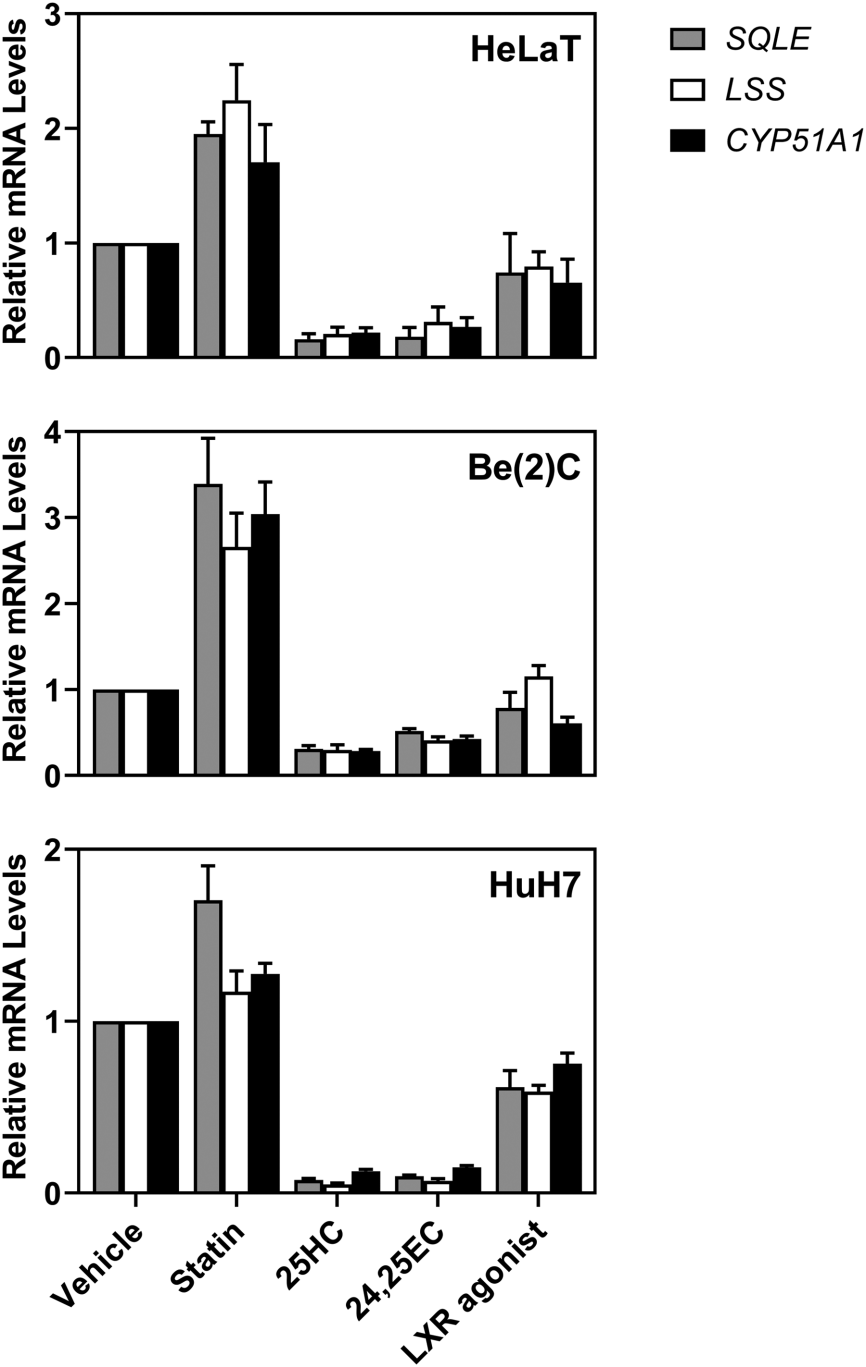
*LSS* and *CYP51A1* gene expression is affected by changing sterol status. HeLaT, HuH7 and Be(2)C cells were treated for 24 h with 5 µM compactin (statin), 10 µM 25-hydroxycholesterol (25HC), 10 µM 24(*S*),25-epoxycholesterol (24,25EC) or 10 µM TO-901317 (LXR agonist) as previously described [46]. *SQLE*, *LSS* and *CYP51A1* mRNA levels were measured using qRT-PCR and normalised to the housekeeping gene *PBGD*. mRNA levels were normalised to vehicle conditions which were set to 1. Data are presented as mean + SEM from at least three independent experiments for each cell line (HeLaT: *n* = 4, Be(2)C: *n* = 3 and HuH7: *n* = 3) each performed in triplicate.

We first investigated the transcriptional regulation of *CYP51A1* and the preceding enzyme, *LSS.* The *LSS* and *CYP51A1* genes have previously been shown to be under the control of SREBP-2 [25,26,43]. To confirm their sterol-responsiveness in HeLaT, HuH7 (liver) and Be(2)C (brain) cells, we treated with statin to decrease sterol status, or with sterols (25-hydroxycholesterol or 24,25-epoxycholesterol) to increase sterol status. We also treated with the liver X receptor (LXR) agonist, TO-901317, as *CYP51A1* is reportedly negatively regulated by LXR [44]. *LSS* and *CYP51A1* displayed similar expression patterns to the established SREBP-2 target gene, *SQLE* [45], under changing sterol conditions (Figure 1). Both *LSS* and *CYP51A1* followed the same trend and were not significantly different from *SQLE*, except HeLaT cells treated with LXR agonist; and HuH7 cells treated with statin, where *LSS* was significantly different from *SQLE*. Therefore, we concluded that in general these three enzymes are transcriptionally regulated in a similar manner.

### LDM protein is rapidly turned over, whilst LSS protein remains stable

**Figure 2.**
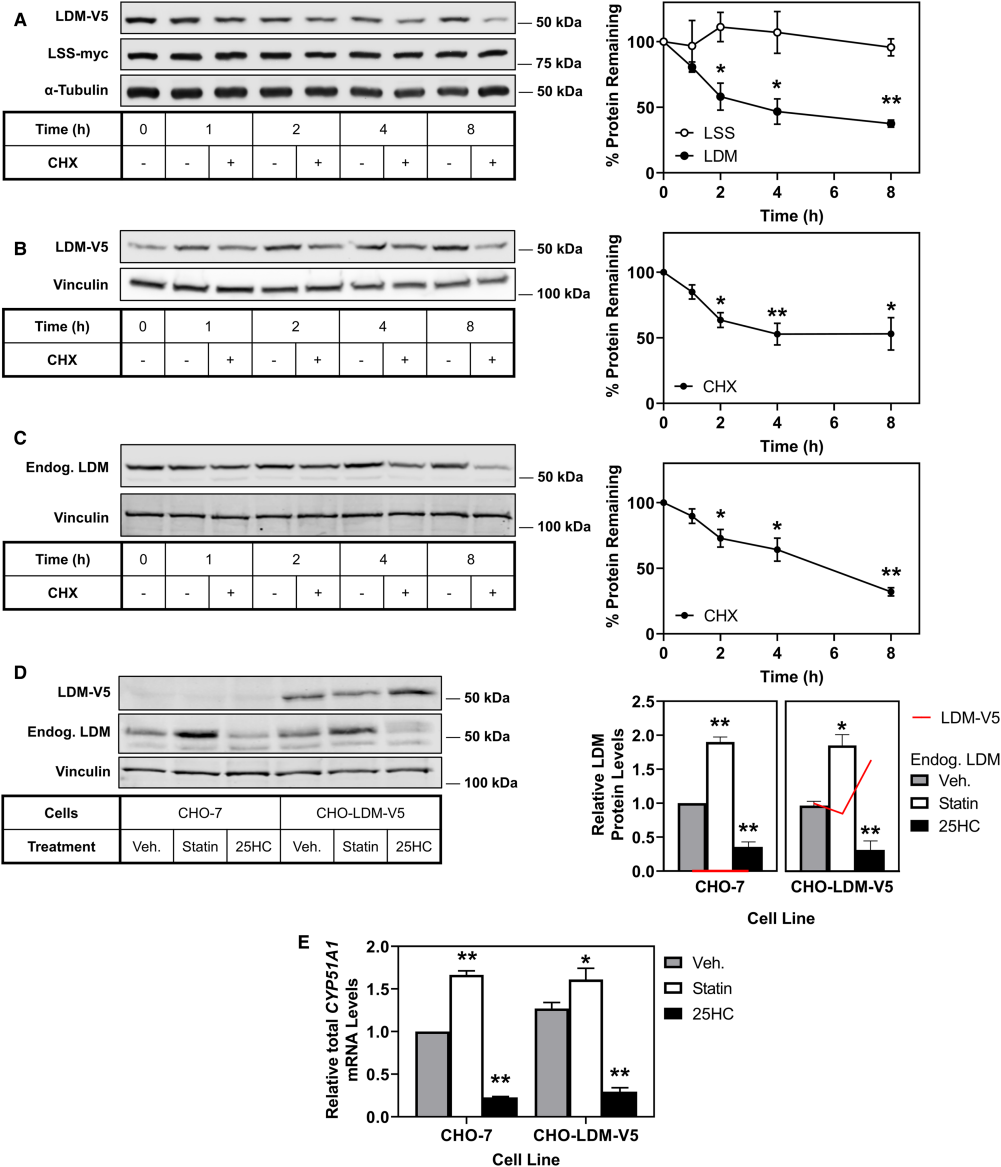
LDM protein is rapidly turned over compared with LSS. (**A**) CHO-LSS-myc cells were transfected with pTK-LDM-V5 plasmid for 24 h and treated with or without 10 µg/ml cycloheximide (CHX) for the indicated time. (**B**) CHO-LDM-V5 cells were treated with or without 10 µg/ml cycloheximide (CHX) for the indicated time. (**C**) CHO-7 cells were treated with or without 10 µg/ml cycloheximide (CHX) for the indicated time. (**D**,**E**) CHO-7 and CHO-LDM-V5 cells were treated with vehicle (Veh), 5 µM compactin (Statin) or 1 µg/ml 25-hydroxycholesterol (25HC) for 24 h. (**D**) Columns are representative of endogenous LDM (*n* = 4) protein levels and red lines are representative of ectopic LDM-V5 (*n* = 1) protein levels. Protein levels were analysed by Western blotting with myc, V5, endogenous LDM, α-tubulin or vinculin antibodies. Total *CYP51A1* mRNA levels were measured using qRT-PCR and normalised to the housekeeping gene *PBGD*. mRNA levels were normalised to vehicle conditions in the CHO-7 cell line which were set to 1. Data are presented as mean ± SEM from at least three independent experiments (**A** *n* = 4, **B** *n* = 3–6, **C** *n* = 4, **D** *n* = 1–4, **E** *n* = 3), where * *P* < 0.05 and ** *P* < 0.01. Relative protein levels were measured using ImageStudio Lite (version 5.2) and normalised to the vehicle condition which was set to 100% (**A-C**) or 1.0 (**D,E**).

We next sought to determine the turnover of LSS and LDM protein. Previously, we found that although DHCR24 and DHCR7 are transcriptionally regulated in a very similar way [46,47], the two terminal enzymes possess vastly different post-translational regulation, with DHCR7 being rapidly turned over, whilst DHCR24 protein is very stable [8]. CHO-7 cells stably expressing LSS with a myc epitope tag (CHO-LSS-myc) [41] were transiently transfected with an LDM-V5 plasmid for 24 h, then treated with or without the protein synthesis inhibitor cycloheximide. LSS-myc remained stable while LDM-V5 was significantly turned over by 2 h, with further degradation over 4 and 8 h (Figure 2A). To minimise transfection variability, we next created a CHO-7 cell line stably expressing LDM-V5 (CHO-LDM-V5). We confirmed that the turnover observed in the transient system was transferable to our stable expression system, where LDM was degraded by greater than 50% after 4 h (Figure 2B). To verify that endogenous LDM is similarly degraded, we performed a cycloheximide time course in CHO-7 cells and observed similar degradation of endogenous LDM to that of our overexpression systems (Figure 2C). We then used the CHO-LDM-V5 cell line to further investigate the turnover of LDM.

### Ectopic LDM differentiates between transcriptional and post-translational effects

The advantage of an ectopic cell line expressing LDM (CHO-LDM-V5) is that it avoids regulation at the transcriptional level by sterols (Figure 1). In these cells, when we manipulate cell sterol status, we observe that levels of endogenous LDM (Figure 2D) reflect transcript levels (Figure 2E), increasing with statin treatment and decreasing with 25-hydroxycholesterol, whereas ectopic LDM did not (Figure 2D). It is also worth noting that CHO-7 and CHO-LDM-V5 contain comparable levels of LDM at both the protein (Figure 2D) and transcript levels (Figure 2E). Therefore, the CHO-LDM-V5 cells represent an ectopically low-expressing rather than overexpressing cell line.

### LDM is not degraded in response to sterols

**Figure 3.**
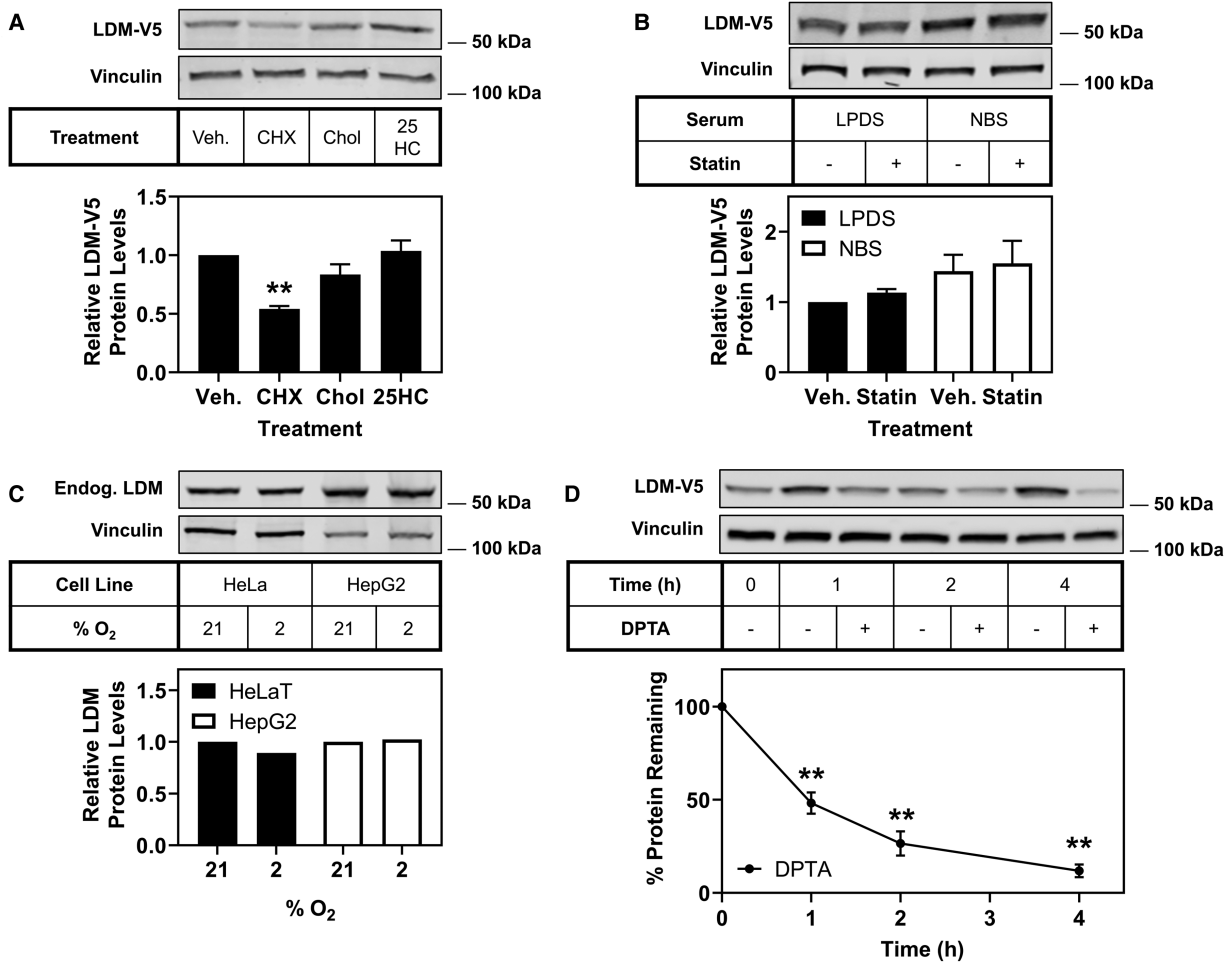
Nitric oxide, but not sterols, trigger LDM for degradation. (**A**) CHO-LDM-V5 cells were treated with 10 µg/ml cycloheximide (CHX), 20 µg/ml cholesterol/CD (Chol) or 1 µg/ml 25-hydroxycholesterol (25HC) for 8 h. (**B**) CHO-LDM-V5 cells were treated in 5% (v/v) LPDS or NBS based media with or without statin (5 µM compactin and 50 µM mevalonate) for 16 h. (**C**) HeLaT and HepG2 cells were exposed to 21% (normoxic) or 2% (hypoxic) oxygen conditions for 4 h; the lysates were obtained from Novus Biologicals. (**D**) CHO-LDM-V5 cells were treated with or without 500 µM DPTA NONOate (DPTA) for the indicated time. Protein levels were analysed by Western blotting with V5, endogenous LDM and vinculin antibodies. Except in (**C**), data are presented as mean ± SEM from at least three independent experiments (**A** *n* = 3, **B** *n* = 5, **D** *n* = 4), where ** *P* < 0.01. Relative protein levels were measured using ImageStudio Lite (version 5.2) and normalised to the vehicle (Veh) or control condition in each blot, which was set to 1 (**A–C**) or 100% at each time point (**D**).

We considered whether LDM is degraded in a similar manner to other cholesterol synthesis enzymes. Some are triggered for degradation by cholesterol (e.g. SM [7] and DHCR7 [8]), oxysterols (e.g. HMGCR [48]), or other cholesterol synthesis intermediates [7,8,49]. When CHO-LDM-V5 cells were treated with cholesterol or 25-hydroxycholesterol, LDM-V5 remained stable (Figure 3A). To test whether other sterol intermediates trigger the degradation of LDM, cells were subjected to treatment with or without statin (compactin) to inhibit the cholesterol synthesis pathway in either lipoprotein-deficient serum (LPDS) or full serum (NBS) media. LDM-V5 did not significantly respond to statin in either serum, with no decrease of LDM-V5 in NBS compared with LPDS (Figure 3B). This indicates sterols are unlikely to participate in the degradation of LDM.

### LDM degradation is not triggered by hypoxia

Since LDM was not degraded in response to sterols, we considered other factors that may trigger its degradation. LDM is one of the most oxygen intensive steps in the cholesterol synthesis pathway, and hypoxia has been shown to accumulate substrates of LDM [50]. We therefore tested whether oxygen depletion would result in the degradation of LDM. We compared LDM levels in normoxic (21% oxygen) and hypoxic (2% oxygen) commercial lysates from HeLaT and HepG2 cells (Novus Biologicals). LDM levels did not change in response to hypoxia in either cell line (Figure 3C), although LDM has been shown to be transcriptionally responsive to hypoxia [27].

### Nitric oxide accelerates the degradation of LDM

During the course of our work, another study found that nitric oxide stimulates the degradation of LDM [28]. We confirmed that LDM-V5 is susceptible to degradation in our system when subjected to treatment with a nitric oxide donor, DPTA NONOate (DPTA), in a time-dependent manner, with more than 50% turnover observed at 1 h and increased turnover at 2 and 4 h (Figure 3D).

### The E3 ubiquitin ligase MARCH6 targets LDM for degradation

**Figure 4.**
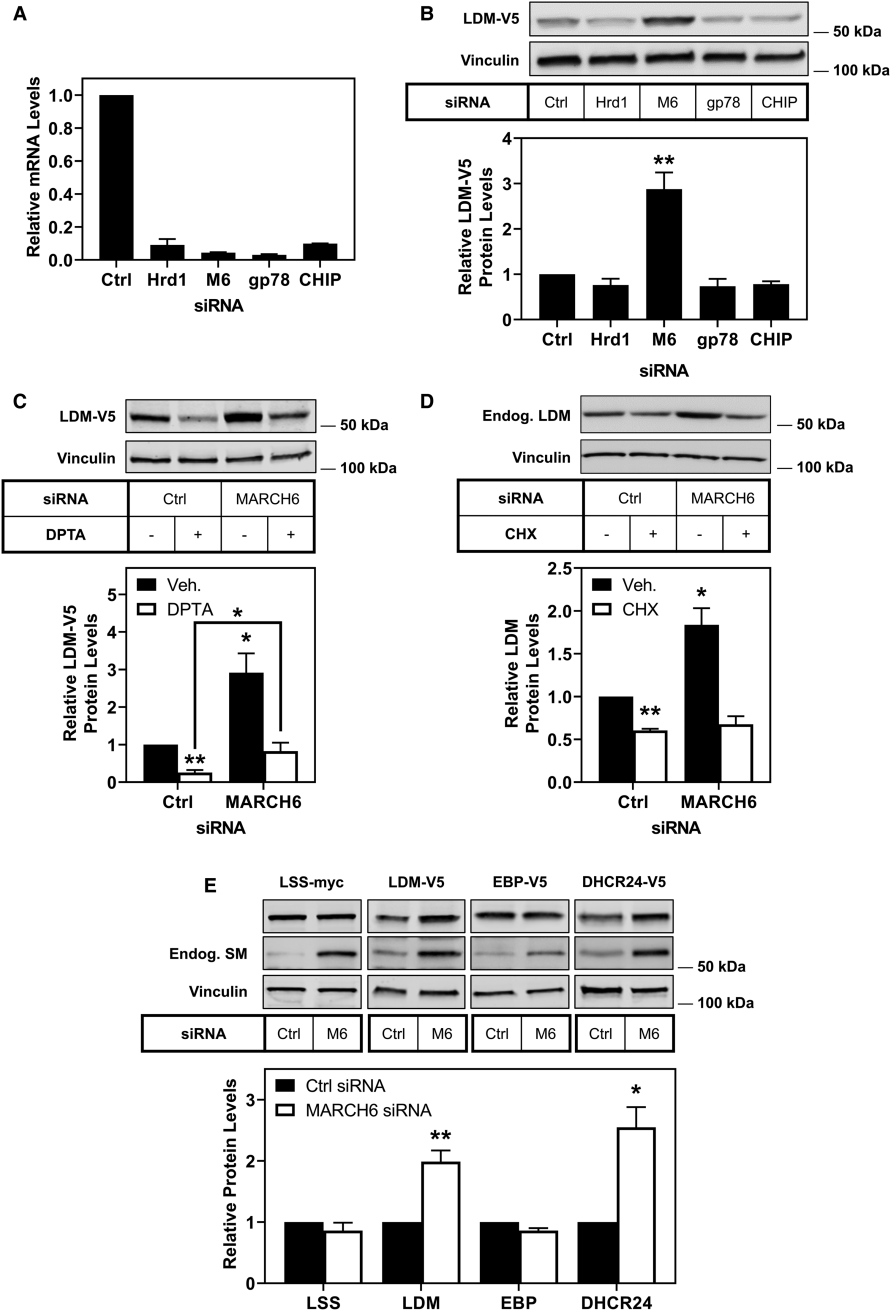
LDM and DHCR24 are degraded by the E3 ubiquitin ligase MARCH6. (**A**) CHO-LDM-V5 cells were transfected for 24 h with 25 nM of the indicated siRNA (M6: MARCH6), then mRNA levels were measured using qRT-PCR and normalised to the housekeeping gene *PBGD*. mRNA levels were normalised to the control condition which was set to 1. Data are presented as mean + half range from an experiment performed in triplicate. (**B**) CHO-LDM-V5 cells were transfected for 24 h with 25 nM of the indicated siRNA (M6: MARCH6). (**C**) CHO-LDM-V5 cells were transfected for 24 h with 25 nM control or MARCH6 siRNA, then treated with or without 500 µM DPTA NONOate (DPTA) for 2 h. (**D**) HepG2 cells were transfected for 24 h with 25 nM control or MARCH6 siRNA, then treated with or without 10 µg/ml cycloheximide (CHX) for 8 h. (**E**) CHO-LSS-myc, CHO-LDM-V5, CHO-EBP-V5 or CHO-DHCR24-V5 cells were transfected for 24 h with 25 nM control or MARCH6 (M6) siRNA. Bands for the ectopic cholesterol synthesis enzymes migrated close to the expected size; LSS-myc: 83 kDa, LDM-V5: 57 kDa, EBP-V5: 26 kDa, DHCR24-V5: 60 kDa. Protein levels were analysed by Western blotting with V5, myc, endogenous SM, endogenous LDM and vinculin antibodies. Data are presented as mean + SEM from at least three independent experiments (**B** *n* = 3–7, **C** *n* = 6, **D** *n* = 3, **E** *n* = 3–6), where * *P* < 0.05 and ** *P* < 0.01. Relative protein levels were measured using ImageStudio Lite (version 5.2) and normalised to the control condition which was set to 1.

The turnover of LDM is likely mediated via the proteasome [28], and so we next considered candidate E3 ubiquitin ligases. Hrd1 and MARCH6 are two major endoplasmic reticulum-associated degradation E3 ubiquitin ligases implicated in the degradation of sterol synthesis enzymes [31,51], and gp78 and CHIP are implicated in the degradation of cytochrome P450s [33]. We used siRNA to knock down each of these E3 ubiquitin ligases (Figure 4A) and measured LDM-V5 protein levels (Figure 4B). Only the knockdown of *MARCH6* resulted in a significant increase in LDM-V5 protein levels (Figure 4B), indicating that MARCH6 is likely an E3 ubiquitin ligase for LDM.

### The nitric oxide triggered degradation of LDM does not require MARCH6

We investigated whether the nitric oxide triggered turnover of LDM was occurring through MARCH6. CHO-LDM-V5 cells were transfected with control or MARCH6 siRNA and then treated with DPTA. LDM-V5 levels were increased with *MARCH6* knockdown, but the nitric oxide-mediated degradation of LDM was not blunted (Figure 4C). There was a significant increase in LDM-V5 levels in the DPTA condition with *MARCH6* knockdown compared with DPTA treatment alone. However, this rescue returned LDM-V5 to basal levels. This indicates that MARCH6 is not solely responsible for the nitric oxide-dependent degradation of LDM.

### MARCH6 regulates LDM in a liver cell line

To confirm that our findings on LDM protein are physiologically relevant, we employed HepG2 cells, a liver cell-line. We found that LDM protein levels were decreased following CHX treatment, and knockdown of *MARCH6* with siRNA increased LDM levels (Figure 4D). Similar to our findings with DPTA, treatment with CHX in the presence of *MARCH6* knockdown still decreased LDM protein levels. This suggests that other E3 ligases may also be involved in the degradation of LDM when MARCH6 is not available.

### The E3 ubiquitin ligase MARCH6 controls levels of LDM and DHCR24

We have previously found that MARCH6 controls the abundance of two cholesterol synthesis enzymes (HMGCR and SM [31]), but not DHCR7 [8]. In this study, we have identified a new MARCH6 substrate in the cholesterol biosynthesis pathway, LDM. We hypothesised that MARCH6 may also control levels of other cholesterol biosynthesis enzymes, especially considering that its own levels are also controlled by cholesterol [37]. We tested LSS, the enzyme located between SM and LDM; DHCR24, a terminal enzyme that also controls entry into the Kandutsch–Russell pathway; and EBP, which is at the branch point to the modified Kandutsch–Russell pathway. The knockdown of *MARCH6* resulted in no change to protein levels of LSS and EBP (Figure 4E). However, the knockdown of *MARCH6* resulted in a 2- or 2.5-fold increase in LDM and DHCR24 levels, respectively (Figure 4E). This indicates that MARCH6 targets LDM and DHCR24 for degradation, but not LSS nor EBP. Additionally, we found that treating cells with the proteasomal inhibitor MG132 resulted in higher molecular mass banding for DHCR24, indicative of increased ubiquitination (Supplementary Figure S1). Although we did not observe higher molecular mass banding for LDM-V5 following this treatment, it should be noted that ubiquitinated peptides of both LDM and DHCR24 have been reported [52].

### LDM and DHCR24 are ubiquitinated and interact with MARCH6

**Figure 5.**
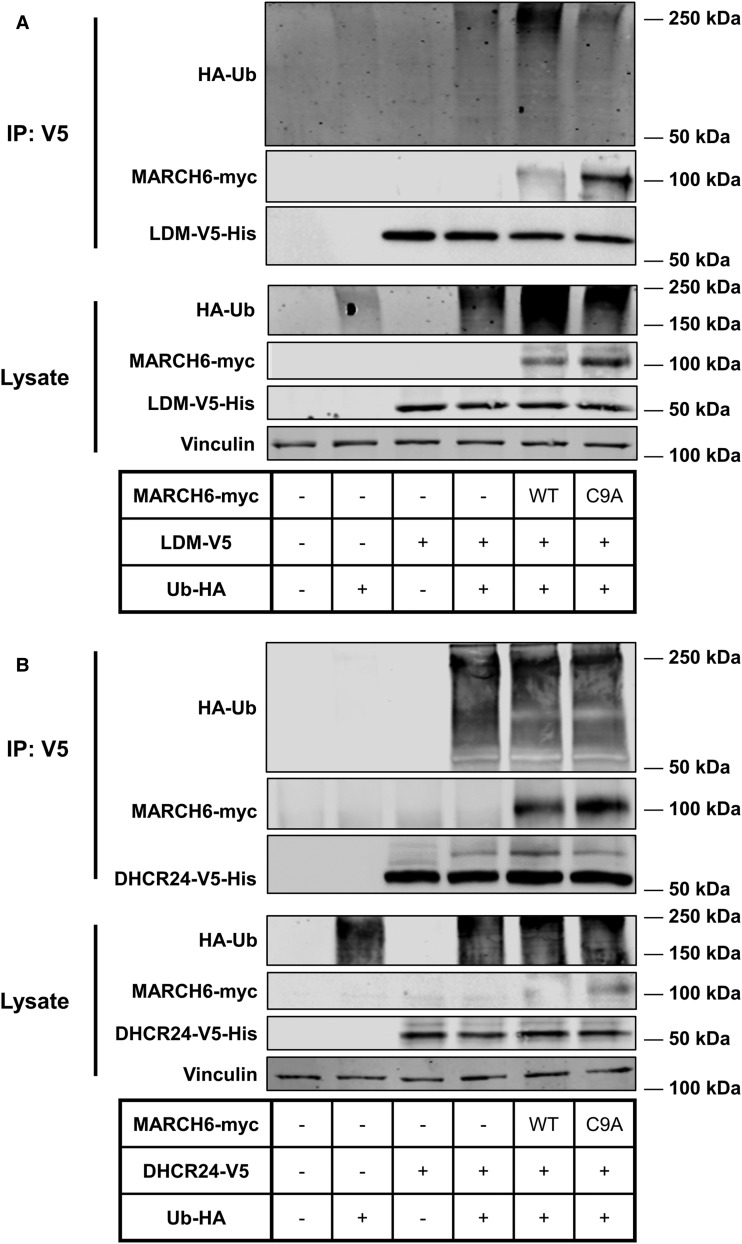
LDM and DHCR24 are ubiquitinated and interact with MARCH6. (**A**,**B**) CHO-7 cells were seeded then co-transfected the next day for 24 h with 5.8 μg DNA (1 : 2 : 2 ratio of HA-Ub:MARCH6-myc:LDM-V5 or DHCR24-V5), then treated for 6 h with 50 μM MG132 and immunoprecipitated (IP) using V5 antibody. Protein levels were analysed by Western blotting with Penta-His, V5, myc, vinculin and HA antibodies. Data are representative of two independent experiments.

Since MARCH6 controlled the levels of LDM and DHCR24, we next tested whether MARCH6 interacts with these proteins. We co-transfected LDM-V5 or DHCR24-V5 with a functional (WT) or non-functional (C9A) MARCH6, and HA-Ubiquitin, and immunoprecipitated with an antibody against V5 (Figure 5). Both MARCH6-WT and MARCH6-C9A were pulled down with LDM-V5 and DHCR24-V5, indicating a physical interaction between the E3 ubiquitin ligase and the substrates. Being non-functional, MARCH6-C9A cannot degrade itself and hence accumulates. However, this mutant E3 ligase can still interact with its substrates, as we have previously found for SM [31]. Furthermore, both LDM-V5 and DHCR24-V5 were ubiquitinated. Of note, a band that migrated ∼8 kDa above DHCR24-V5, consistent with mono-ubiquitination, was more intense in the MARCH6-WT immunoprecipitation compared with the other lanes (Figure 5B). Taken together, our immunoprecipitation experiments provide additional evidence for the role of MARCH6 in the ubiquitination of LDM and DHCR24.

## Discussion

In this study, we identified LDM and DHCR24 as two new MARCH6 substrates. Firstly, we demonstrated that *LSS* and *CYP51A1* [25,26,43], like most cholesterol synthesis genes, are sterol-responsive (Figure 1). Secondly, we demonstrated that LSS and LDM undergo distinct post-translational regulation. LSS is a stable protein, whereas LDM is more labile (Figure 2) and undergoes degradation triggered by nitric oxide (Figure 3D). However, LDM does not respond post-translationally to sterols (Figure 3A,B) like some other cholesterol synthesis enzymes [5–8]. Furthermore, we identified MARCH6 as an E3 ubiquitin ligase involved in the degradation of LDM, and also implicated MARCH6 in the degradation of another cholesterol synthesis enzyme, DHCR24 (Figures 4, 5).

Like most cholesterol synthesis enzymes, the genes *LSS* and *CYP51A1* are regulated through changing sterol status (Figure 1) by SREBP-2, a master transcription factor controlling the pathway. However, *CYP51A1* possesses additional layers of transcriptional regulation via cyclic-AMP [25,26] and hypoxia [27]. A negative LXR element has also been suggested [44], but we did not observe a consistent significant change in *CYP51A1* levels following treatment with an LXR agonist (Figure 1).

Beyond transcriptional regulation, we investigated the post-translational regulation of LSS and LDM. We have previously characterised pre-lanosterol (SM [7,31,53–56]) and the terminal (DHCR7 [8,47,57,58] and DHCR24 [9,46,59]) enzymes of cholesterol synthesis. However, little research has been conducted on the intermediate enzymes within the pathway. LSS immediately follows SM, forming the first sterol intermediate in the pathway (lanosterol), and LDM acts as a gateway enzyme to the post-lanosterol pathway. We found distinct post-translational regulation of these enzymes, which would likely result in an accumulation of intermediates under various conditions. Furthermore, the substrates of LDM, lanosterol and 24,25-dihydrolanosterol, have key regulatory functions including triggering degradation of the rate-limiting enzyme HMGCR [60] and modulating immune responses [61]. Therefore, the stability of LSS likely pushes flux from 2,3-oxidosqualene to lanosterol rapidly and promotes feedback regulation via accumulation of lanosterol and 24,25-dihydrolanosterol. By LDM being highly regulated, it can act as a switch to down-regulate or up-regulate either the Bloch or Kandutsch–Russell pathway.

Remarkably, unlike other cholesterol synthesis enzymes that are degraded by increased sterol levels, LDM remained stubbornly stable (Figure 3A,B). This is similar to Erg11p (yeast LDM), which also demonstrates a lack of degradation in response to changing sterol levels by inhibition of enzymes in the pathway [30]. Since LDM was rapidly degraded, we instead considered an alternative trigger that may play a role in its degradation. We tested whether hypoxia triggered the degradation of LDM, as oxygen is a potential limiting factor, however, LDM protein levels remained unchanged (Figure 3C).

During the course of our work, nitric oxide was identified as a trigger for the degradation of LDM likely via the proteasome [28], much like other cytochrome P450s [62,63]. However, the mechanism for degradation of LDM is complex, with Park et al. [28] reporting degradation of LDM involving both a proteasomal component and possibly calpains. We similarly found complex degradation mechanisms for LDM, in that whilst MARCH6 plays a clear role in ubiquitinating and degrading LDM (Figures 4, 5), it is probably not the only E3 ligase involved (Figure 4C,D).

We confirmed that nitric oxide can trigger the degradation of LDM in our experimental system (Figure 3D). Nitric oxide has been linked to cholesterol previously, with its role seemingly protective by reducing circulating cholesterol and the risk of atherosclerotic lesions [64]. Furthermore, nitric oxide is responsible for numerous protein modifications including: oxidation of thiols in cysteine residues [65], nitration of tyrosine residues [66] and ligation to the heme group [67]. Whether nitric oxide causes an indirect change through signalling cascades or direct protein modification on LDM could be explored in future work.

We identified that the E3 ubiquitin ligase MARCH6 is responsible for the degradation of LDM. MARCH6 plays a key role in the degradation of cholesterol — and other lipid metabolism-related proteins [31,34–36]. Furthermore, MARCH6 itself is stabilised in high sterol conditions, allowing for an increase in activity and rapid shut down of processes including cholesterol synthesis [37]. It is currently not clear why cholesterol stabilises MARCH6 protein and promotes degradation of SM [37], but cholesterol does not affect LDM or DHCR24 levels, despite MARCH6's involvement in their degradation. It is likely that additional regulation mechanisms are involved. For example, we have found that SM undergoes a conformational change in response to cholesterol [54,55], which may be required in concert with the increase in MARCH6 levels for robust regulation to occur.

Although MARCH6 plays a role in the basal control of LDM levels (Figure 4B), degradation of LDM triggered by nitric oxide was not dependent on MARCH6 (Figure 4C), suggesting the involvement of other E3 ligases and/or degradation pathways. This is the first time that MARCH6 has been implicated in the degradation of a cytochrome P450, and MARCH6 could potentially play an important role in the regulation of other proteins of that class.

MARCH6 also regulates the cholesterol synthesis enzymes HMGCR (for which several E3 ubiquitin ligases have been identified [51]) and SM [31], but not DHCR7 [8], and so we tested whether MARCH6 might be involved in the degradation of other enzymes within the pathway. LSS and LDM are differently regulated post-translationally and MARCH6 plays a role in controlling the levels of LDM but not LSS (Figure 4E). We also tested whether MARCH6 affects DHCR24, which is the gateway enzyme to the Kandutsch–Russell pathway, the terminal enzyme in the Bloch pathway and can theoretically act on any intermediate in the Bloch pathway to transfer to the Kandutsch–Russell pathway [10]. Surprisingly, this typically stable yet highly regulated enzyme is also controlled by MARCH6 (Figure 4E) [68]. We found that MARCH6 interacts with LDM and DHCR24 and both enzymes are ubiquitinated (Figure 5).

**Figure 6.**
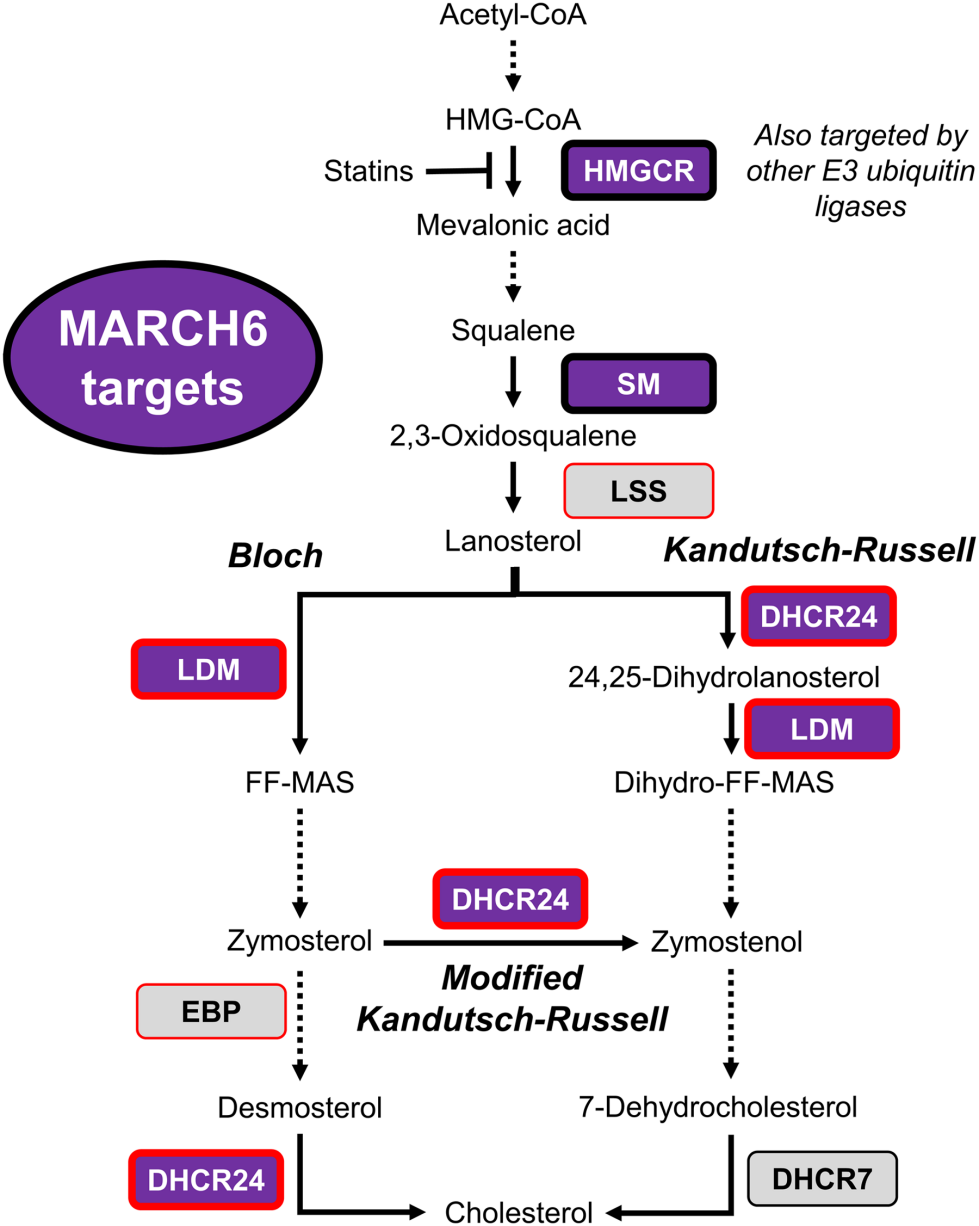
Simplified schematic of the cholesterol biosynthesis pathway and MARCH6 targets. The cholesterol biosynthesis pathway contains over 20 enzymes and multiple branch points have been suggested between the Bloch and Kandutsch–Russell pathway. MARCH6 targets are indicated in purple, and enzymes that are not MARCH6 targets in grey, with discoveries from this paper outlined in red.

Although sterols have no effect on DHCR24 protein levels [39], the steroid hormone pregnenolone, and the tyrosine kinase inhibitors masitinib and ponatinib decrease DHCR24 protein levels [69]. DHCR24 enzyme activity can also be decreased by progestin steroid hormones (progesterone and pregnenolone) [70] and phosphorylation of tyrosine residues [9]. This suggests that DHCR24 is highly regulated in a variety of ways, and more work is needed to elucidate this further. EBP, the other enzyme at the branch point to the modified Kandutsch–Russell pathway (Figure 6), does not appear to be a MARCH6 substrate (Figure 4E). This multi-targeted shutdown of cholesterol synthesis by a single E3 ligase (Figure 6), likely means that only certain intermediates accumulate within the pathway. For instance, degradation of LDM would likely result in a build-up of lanosterol, which has several important roles, such as triggering degradation of the rate-limiting enzyme HMGCR [60] and modulating immune responses [61].

In conclusion, *LSS* and *CYP51A1* are regulated transcriptionally in a similar manner via sterols, but LSS and LDM undergo distinct post-translational regulation. LDM does not respond to sterols but is turned over in response to nitric oxide, and probably other triggers yet to be discovered. Degradation of LDM occurs, at least in part, via the E3 ubiquitin ligase MARCH6, which may additionally play a role in the degradation of DHCR24. Remarkably, MARCH6 is the first example of an E3 ubiquitin ligase that targets multiple steps in a single biochemical pathway. Overall, this indicates novel inputs into the control of cholesterol synthesis along the length of the pathway.

**Note added in proof 22 January 2020:** In further work, we have just published that another cholesterol synthesis enzyme, DHCR14, is not similarly targeted by MARCH6 [46].

## Abbreviations

CHO-7: Chinese hamster ovary-7
CYP51A1: cytochrome P450 family 51 subfamily A member 1
DHCR24: 24-dehydrocholesterol reductase
DHCR7: 7-dehydrocholesterol reductase
EBP: emopamil-binding protein
FCS: foetal calf serum
FF-MAS: follicular fluid meiosis-activating sterol
HMGCR: 3-hydroxy-3-methylglutaryl coenzyme A reductase
LDM: lanosterol 14α-demethylase
LPDS: lipoprotein-deficient newborn calf serum
LSS: lanosterol synthase
LXR: liver X receptor
MARCH6: membrane-associated ring-CH-type finger 6
SM: squalene monooxygenase
SREBP-2: sterol-regulatory element-binding protein-2

## Open Access

Open access for this article was enabled by the participation of University of New South Wales in an all-inclusive *Read & Publish* pilot with Portland Press and the Biochemical Society under a transformative agreement with CAUL.
