# Supplementary material for: The cholesterol synthesis enzyme lanosterol 14α-demethylase is post-translationally regulated by the E3 ubiquitin ligase MARCH6
Source: Biochem J. 2020 Jan 31;477(2):541–55. doi: 10.1042/BCJ20190647 (PMC6993871; doi:10.1042/BCJ20190647)

Supplemental Data

Supplementary Figure 1. DHCR24 but not LDM accumulates high molecular weight products during proteasomal inhibition. CHO-LDM-V5 or CHO-DHCR24-V5 cells were treated with or without 50 μM MG132 for 8 h. Protein levels were analysed by Western blotting with V5 and vinculin antibodies. Data are representative of two independent experiments.

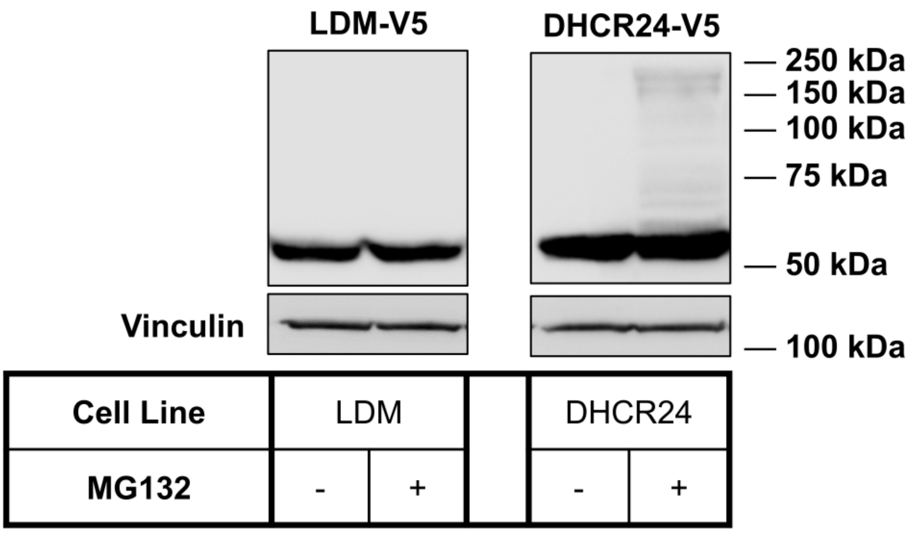

Supplement: Supplementary Figure S1 [file BCJ-477-541-s1.pdf]
